# Association between estimated plasma volume status and risk of sepsis-associated acute kidney Injury: a retrospective cohort study using the MIMIC-IV database

**DOI:** 10.1080/0886022X.2025.2586382

**Published:** 2025-11-14

**Authors:** Yifeng Chen, Mingkun Yang, Yanwen Lu, Xindian Pan, Xiaofeng Zhang, Shenghui Miao, Jing Yan, Weihang Hu

**Affiliations:** ^a^ The Second School of Clinical Medicine, Zhejiang Chinese Medical University, Hangzhou, Zhejiang, China; ^b^ Zhejiang Hospital, Hangzhou, Zhejiang, China; ^c^ Department of Pathology, Wenzhou People’s Hospital, Wenzhou, Zhejiang, China; ^d^ Department of Critical Care medicine, The First People’s Hospital of Aksu Region, Aksu, Xinjiang, China; ^e^ Department of Critical Care medicine, The First Affiliated Hospital of Zhejiang Chinese Medical University (Zhejiang Provincial Hospital of Traditional Chinese Medicine), Hangzhou, Zhejiang, China; ^f^ Department of Critical Care medicine, Zhejiang Hospital, Hangzhou, Zhejiang, China

**Keywords:** Estimated plasma volume status, sepsis-associated acute kidney Injury, MIMIC-IV, prognosis

## Abstract

Estimated plasma volume status (ePVS) is a simple, useful tool for assessing volume status. However, its prognostic value in sepsis-associated acute kidney injury (SA-AKI) remains unclear. This retrospective cohort study analyzed data from 15,789 patients with SA-AKI extracted from the MIMIC-IV database. The association between ePVS and mortality was evaluated using Cox regression and Kaplan-Meier analysis. Logistic regression was employed to assess the relationship between ePVS and renal recovery. All models were adjusted for demographics, comorbidities, laboratory values, and illness severity scores. Restricted cubic spline (RCS) model was applied to examine potential nonlinear relationships between ePVS and mortality risk. Among 15,789 eligible patients with SA-AKI, the 28-day mortality rate was 23.2%. Multivariate Cox regression analysis revealed that higher ePVS was associated with increased 28-day mortality (HR = 1.05, 95% CI 1.03–1.07, *p* < 0.001) and logistic regression analysis further indicated that elevated ePVS was correlated with reduced renal recovery (OR = 0.96, 95% CI: 0.94–0.99, *p* = 0.002). RCS demonstrated a nonlinear relationship between ePVS and mortality, with an inflection point identified at 5.47 dL/g. Using this cutoff, the high-ePVS group exhibited significantly higher 28-, 60-, and 180-day mortality and lower renal recovery than the low-ePVS group. ePVS is an independent risk factor for 28-day mortality in SA-AKI, showing a U-shaped association. These findings suggest ePVS may serve as a useful prognostic marker to guide fluid management strategies in SA-AKI.

## Introduction

Sepsis-associated acute kidney injury (SA-AKI) is a frequent and serious complication of sepsis, markedly elevating mortality rates and presenting a major challenge in critical care management [1,2]. The pathogenesis of sepsis is intricately linked to insufficient effective blood volume, endothelial cell dysfunction, and the leakage of fluid into the interstitial space [3]. Traditionally, the management of SA - AKI has focused on optimizing blood pressure to enhance renal perfusion pressure, thereby maintaining renal perfusion and function [4]. However, previous studies have indicated that a positive fluid balance is associated with worse outcomes in patients with SA - AKI [5–7]. Therefore, accurately assessing the initial volume status of patients with SA - AKI is crucial to prevent volume overload during fluid resuscitation.

Current methods for assessing volume status in patients with SA-AKI, such as physical examination, central venous pressure monitoring, and chest X-ray, which are often imprecise and subjective [8,9]. Although point-of-care ultrasound has improved assessment accuracy, its clinical utility remains limited by operator dependence and inconsistent availability [10]. Plasma volume (PV) plays a crucial role in maintaining the balance between interstitial and intravascular compartments, acting as a key marker of volume overload [11,12]. By estimating PV and dynamically monitoring it over time, clinicians can assess a patient’s volume status, allowing for timely adjustments to the fluid management plan [13,14]. Despite being the gold standard, radioisotope dilution techniques for plasma volume quantification are unsuitable for routine clinical use due to high cost and prolonged processing time [15,16]. These limitations underscore the difficulty in obtaining frequent and reliable PV measurements in practice. Therefore, there is an urgent need for an objective, easily accessible, and quantifiable metric to accurately detect volume overload and guide fluid management.

To address these limitations, Strauss et al. [17] developed an equation based on hematocrit and hemoglobin to estimate plasma volume status (ePVS), offering a low-cost and rapid alternative. Duarte et al. [18] further extended Strauss’s formula, enabling instantaneous PV estimation using hematocrit and hemoglobin data from a single time point. ePVS has been shown to correlate with adverse cardiovascular outcomes in patients with various types of heart failure [13,18,19]. Beyond heart failure, ePVS has demonstrated prognostic value in patients with acute myocardial infarction [20], after aortic valve replacement [21], lower extremity artery disease [22], and in those with acute respiratory distress syndrome [23].

Given the central role of volume homeostasis in the pathogenesis and outcomes of SAAKI, we hypothesized that ePVS at intensive care unit (ICU) admission would be independently associated with prognostic risk in SAAKI patients. This study investigates the correlation between ePVS and prognostic risk in SAAKI patients, aiming to provide a simple, effective prognostic tool and insights for guiding their fluid management strategies.

## Materials and methods

### Data sources

The MIMIC - IV database is a single - center, open - access repository that encompasses clinical information from patients hospitalized at Beth Israel Deaconess Medical Center (BIDMC) in Boston, Massachusetts, USA, between 2008 and 2019 [24]. It contains comprehensive data on patient demographics, laboratory results, vital signs, treatments, diagnoses, and follow - up information. Due to the anonymized nature of the database and the absence of protected health information, the BIDMC Institutional Review Board granted a waiver of informed consent, permitting the data to be used for research purposes.

### Population selection

We screened the MIMIC-IV 3.1 database to identify patients who met the diagnostic criteria for SA-AKI, which served as the target population for this study. Sepsis was diagnosed based on the Third International Consensus Definitions for Sepsis and Septic Shock (Sepsis-3.0) [25], defined by a Sequential Organ Failure Assessment (SOFA) score ≥2 and a suspected or confirmed infection during hospitalization. Acute kidney injury (AKI) was diagnosed according to the Kidney Disease Improving Global Outcomes (KDIGO) creatinine criteria [26], which include either: (1) a decrease in urine output to <0.5 mL/kg/h for ≥6 h, or (2) a serum creatinine increase of ≥0.3 mg/dL within 48 h or *a* ≥ 1.5-fold increase from the baseline, known or presumed to have occurred within the prior 7 days. Exclusion criteria included: (a) an ICU length of stay <2 days, (b) age <18 years, or (c) missing hematocrit or hemoglobin data within 24 h prior to admission. For patients with multiple ICU admissions or hospitalizations during the same stay, only data from the first ICU admission were included.

### Data collection

We utilized Structured Query Language (SQL) and PostgreSQL (version 9.6) to extract data on ICU patients from the MIMIC - IV database. The extracted variables encompassed demographic characteristics (age, sex, weight, race), vital signs (body temperature, heart rate, respiratory rate, systolic blood pressure, diastolic blood pressure, peripheral oxygen saturation SpO_2_), and laboratory examination results (white blood cell count (WBC), hemoglobin, hematocrit, platelet, sodium, potassium, calcium, chloride, glucose, serum creatinine, blood urea nitrogen (BUN), prothrombin time (PT), activated partial thromboplastin time (APTT)). Comorbidities taken into account included heart failure, chronic obstructive pulmonary disease (COPD), cancer, diabetes, and kidney disease. The severity of the disease was assessed using the Sequential Organ Failure Assessment (SOFA) score, Simplified Acute Physiology Score II (SAPSII), and Glasgow Coma Scale (GCS). Management within the first 24 h prior to ICU admission included renal - replacement therapy (RRT) and vasoactive support. Outcome variables comprised 28 - day, 60 - day, and 180 - day mortality, as well as the rate of renal function recovery. Renal function recovery is defined as a patient’s KDIGO stage regressing to stage 0 at the final follow-up, with 28 - day mortality serving as the primary outcome. The ePVS score was calculated using the formula: (100 - hematocrit (%))/hemoglobin (g/dL) [15].

### Statistical analysis

In terms of statistical analysis, continuous variables were summarized in the form of median and interquartile range (IQR) and compared using the Wilcoxon rank - sum test. Categorical variables were presented as counts and percentages and compared using the chi - square test or Fisher ‘s exact test. Cox proportional hazards models were employed to assess the association between ePVS and mortality in SA-AKI patients, while logistic regression models were used to examine the relationship between ePVS and renal function recovery. Both analyses were adjusted for potential confounders. Specifically, the unadjusted model did not include any variable adjustments. Model 1 was adjusted for age, sex, race, weight, AKI stage, use of vasopressor drugs on day 1, use of renal replacement therapy on day 1, and relevant comorbidities (heart failure, COPD, cancer, diabetes, kidney disease). Model 2 further adjusted for laboratory and clinical parameters, including white blood cell count, platelet count, PT, APTT, INR, sodium, potassium, calcium, chloride, glucose, serum creatinine, BUN, heart rate, systolic blood pressure, diastolic blood pressure, respiratory rate, body temperature, blood oxygen saturation, and severity scores (SOFA, SAPSII, GCS), based on Model 1. To assess potential multicollinearity among the covariates in the multivariable models, the variance inflation factor (VIF) was calculated. A VIF value of less than 10 was used to indicate the absence of severe multicollinearity. The relationship between ePVS and 28-day mortality in patients with SA-AKI was assessed using a multivariable-adjusted restricted cubic (RCS) model. The optimal number of knots was selected in order to ensure the model could adequately capture nonlinear associations as well as prevent overfitting. Upon detection of a non-linear association, the optimal threshold was identified using an iterative grid search method, selected to maximize the partial likelihood of a segmented Cox proportional hazards model. Once the ePVS threshold was determined, the cohort was divided into high and low ePVS groups. The Cox proportional hazards model was then applied to evaluate the association between mortality and renal function recovery rates within these two ePVS groups. Kaplan-Meier survival curves were plotted to analyze the cumulative incidence of death. Additionally, exploratory subgroup analyses were conducted to assess the robustness of the correlation between ePVS and prognosis in SA-AKI patients. These subgroups were defined based on the following baseline characteristics: sex, race, diabetes, COPD, cardiovascular disease, kidney disease, malignancy, and SOFA score. All statistical analyses were performed using R version 4.1.2 (R Foundation), with statistical significance set at a two-sided p-value of less than 0.05.

## Result

### Baseline characteristics

**Figure 1.**
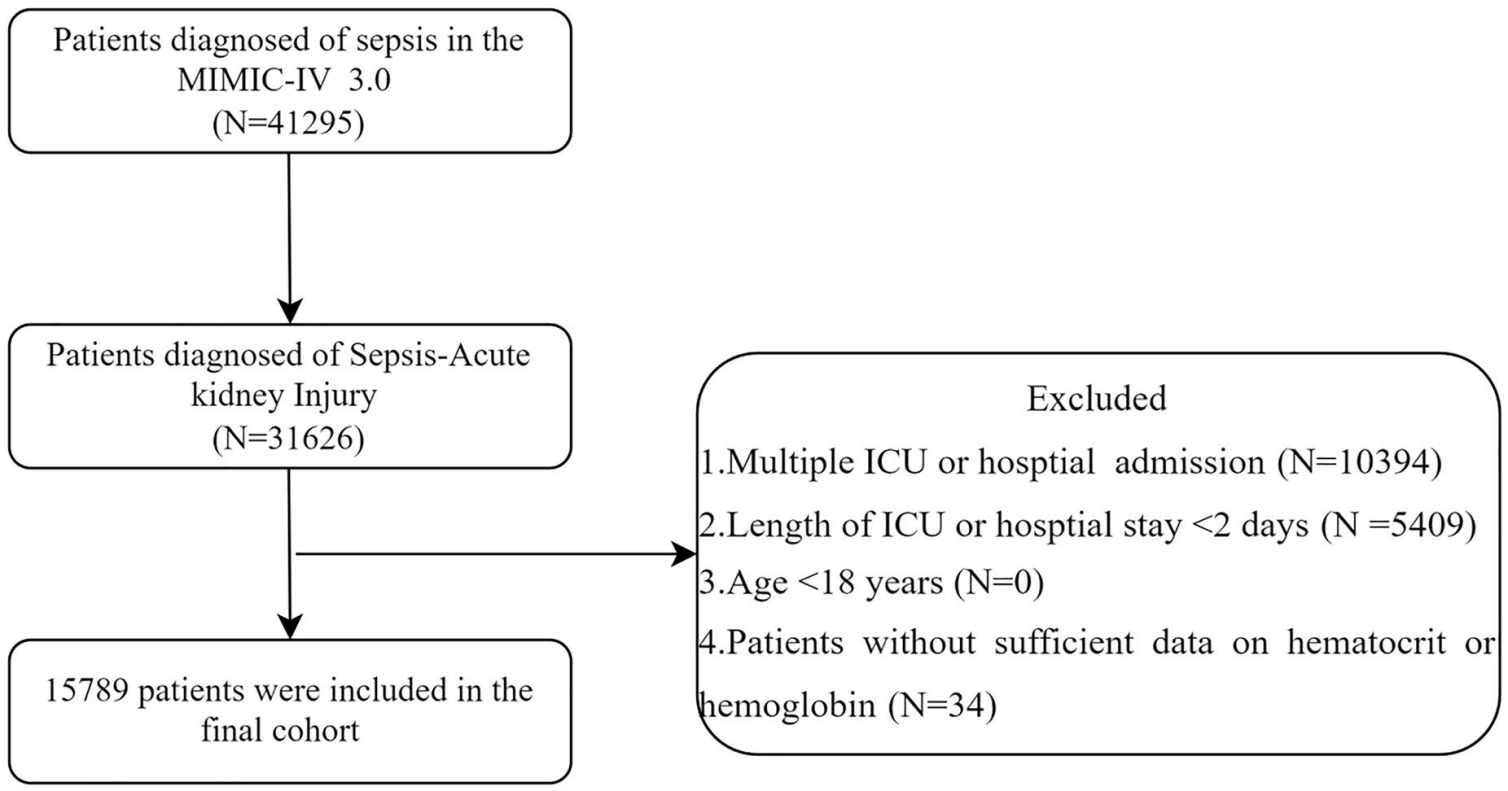
Flowchart of patient selection.

A total of 15,789 patients with SA-AKI were included according to predefined inclusion and exclusion criteria (Figure 1). Table 1 presents the baseline characteristics of patients with SA-AKI. The overall 28-day mortality rate was 23.2%. Among the patients, 6,663 were women (42%), with a median age of 68.25 years (IQR, 56.97–79.03) and a median ePVS of 5.76 dL/g (IQR, 4.67–7.06). Significant differences were observed between the death and survival groups for all baseline variables, except for sex, history of diabetes, and use of vasoactive drugs (*p* < 0.05). Patients in the death group were older and had a higher prevalence of heart failure, COPD, malignancy, and chronic kidney disease compared to the survival group. Additionally, the death group exhibited a higher KDIGO grade. Laboratory analyses revealed significantly elevated WBC, blood glucose levels, INR, PT, APTT, serum sodium, potassium, calcium, BUN, and serum creatinine levels in the death group. Regarding clinical parameters, the death group demonstrated significantly higher heart rates, diastolic blood pressure, respiratory rates, SOFA scores, SAPSII, and GCS scores compared to the survival group.

**Table 1.** Baseline characteristics of patients with SA-AKI.

Characteristic	Overall *N* = 15,789	Survival *N* = 12,132	Death *N* = 3,657	*p*-value [1]
Age	68.25 (56.97–79.03)	67.08 (55.73–77.67)	72.72 (61.42–82.67)	<0.001
Race, *n* (%)				<0.001
White	10,051 (64)	7,905 (65)	2,146 (59)	
Black	1,325 (8.4)	1,050 (8.7)	275 (7.5)	
Other	4,413 (28)	3,177 (26)	1,236 (34)	
Sex, *n* (%)				0.074
Female	6,663 (42)	5,073 (42)	1,590 (43)	
Male	9,126 (58)	7,059 (58)	2,067 (57)	
Weight (kg)	81.50 (68.50–97.50)	82.80 (69.70–98.80)	77.90 (65.00–93.00)	<0.001
Congestive heart failure, *n* (%)	5,186 (33)	3,824 (32)	1,362 (37)	<0.001
COPD, *n* (%)	4,251 (27)	3,212 (26)	1,039 (28)	0.021
Cancer, *n* (%)	2,323 (15)	1,451 (12)	872 (24)	<0.001
Diabetes, *n* (%)	4,997 (32)	3,866 (32)	1,131 (31)	0.28
RRT, *n* (%)	1,056 (6.7)	708 (5.8)	348 (9.5)	<0.001
Vasoactive drug support, *n* (%)	7,260 (46)	5,546 (46)	1,714 (47)	0.22
Renal failure, *n* (%)	3,692 (23)	2,678 (22)	1,014 (28)	<0.001
AKI stage, *n* (%)				<0.001
I	13,213 (84)	10,223 (84)	2,990 (82)	
II	1,846 (12)	1,417 (12)	429 (12)	
III	730 (4.6)	492 (4.1)	238 (6.5)	
WBC (K/uL)	14.30 (10.30–19.40)	14.20 (10.20–19.10)	14.80 (10.50–20.40)	<0.001
Hemoglobin, (g/dL)	11.40 (9.80–13.00)	11.50 (10.00–13.05)	10.90 (9.40–12.70)	<0.001
Hematocrit (%)	34.70 (30.30–39.50)	34.90 (30.60–39.60)	33.90 (29.00–39.10)	<0.001
ePVS (g/dl)	5.76 (4.67–7.06)	5.67 (4.63–6.92)	6.05 (4.80–7.57)	<0.001
Platelets (K/uL)	205.00 (147.00–278.00)	206.00 (151.00–276.00)	202.00 (133.00–284.00)	<0.001
Glucose (mg/dL)	153.00 (123.00–204.00)	150.00 (122.00–197.50)	163.00 (128.00–221.00)	<0.001
INR(s)	1.40 (1.20–1.70)	1.40 (1.20–1.60)	1.50 (1.20–2.00)	<0.001
PT(s)	15.10 (13.20–18.40)	15.10 (13.10–17.60)	16.00 (13.60–22.10)	<0.001
APTT(s)	33.90 (29.00–46.80)	33.90 (28.70–44.10)	36.10 (29.90–56.20)	<0.001
Na (mEq/L)	140.00 (137.00–143.00)	140.00 (137.00–143.00)	140.00 (137.00–144.00)	0.029
K (mEq/L)	4.50 (4.10–5.00)	4.50 (4.10–5.00)	4.60 (4.10–5.20)	<0.001
Ca (mEq/L)	8.50 (8.10–9.00)	8.50 (8.10–8.90)	8.60 (8.10–9.10)	<0.001
Cl (mEq/L)	106.00 (102.00–110.00)	107.00 (102.00–110.00)	105.00 (100.00–110.00)	<0.001
BUN (mg/dL)	24.00 (16.00–41.00)	22.00 (16.00–36.00)	33.00 (21.00–53.00)	<0.001
Creatinine (mg/dL)	1.20 (0.90–2.00)	1.20 (0.80–1.80)	1.50 (1.00–2.50)	<0.001
Heart rate (bpm)	105.00 (91.00–121.00)	104.00 (91.00–119.00)	108.00 (94.00–124.00)	<0.001
SBP (mmHg)	146.00 (133.00–162.00)	147.00 (133.00–163.00)	145.00 (130.00–161.00)	<0.001
DBP (mmHg)	85.00 (74.00–98.00)	84.00 (73.00–98.00)	86.00 (74.00–99.00)	0.006
Respiratory rate (bpm)	28.00 (24.00–32.00)	27.00 (24.00–32.00)	29.00 (25.00–34.00)	<0.001
Temperature (°C)	37.00 (37.00–38.00)	37.00 (37.00–38.00)	37.00 (37.00–38.00)	<0.001
SpO2 (%)	100.00 (100.00-100.00)	100.00 (100.00-100.00)	100.00 (100.00-100.00)	<0.001
SOFA	6.00 (4.00–9.00)	6.00 (3.00–8.00)	7.00 (5.00–10.00)	<0.001
SAPSII	41.00 (33.00–51.00)	39.00 (31.00–48.00)	48.00 (39.00–58.00)	<0.001
GCS	15.00 (13.00–15.00)	15.00 (13.00–15.00)	15.00 (12.00–15.00)	<0.001

^1^ Wilcoxon rank sum test; Pearson’s Chi-squared test.

Continuous variables are expressed in the form of median ± IQR, while categorical variables are presented in the form of number (n) and percentage (%). WBC: White Blood Cell; PT: Prothrombin Time; APTT: Activated Partial Thromboplastin Time; BUN: Blood Urea nitrogen; COPD: Chronic Obstructive Pulmonary Disease; SBP: Systolic blood pressure; DBP: Diastolic blood pressure; SOFA: Sequential Organ Failure Assessment; SAPSII: Simplified Acute Physiology Score II, RRT: Renal Replacement Therapy.

### Correlation between ePVS and prognosis in SAAKI patients

To explore the relationship between ePVS and prognosis in SA-AKI patients, we first performed Cox proportional hazards regression analysis. Multicollinearity diagnostics showed VIFs <10 for all covariates, suggesting no substantive collinearity concerns (Tables 1). As shown in Table 2, ePVS levels were significantly associated with the risk of death at 28, 60, and 180 days. In unadjusted models, ePVS as a continuous variable was significantly associated with 28-day mortality risk (HR 1.12, 95% CI 1.10–1.14, *p* < 0.001). After adjusting for potential confounders, the association remained significant in both Model 1 (HR 1.07, 95% CI 1.05–1.09, *p* < 0.001) and Model 2 (HR 1.05, 95% CI 1.03–1.07, *p* < 0.001). Similar associations were observed for 60-day and 180-day mortality.

**Table 2.** Association between ePVS and prognostic outcomes in patients with SA-AKI.

	Crude model		Model 1		Model 2	
	95%CI	*P*	95%CI	*P*	95%CI	*P*
28-day mortality						
ePVS	1.12 (1.10–1.14)	<0.001	1.07 (1.05–1.09)	<0.001	1.05 (1.03–1.07)	<0.001
60-day mortality						
ePVS	1.13 (1.11–1.15)	<0.001	1.08 (1.06–1.10)	<0.001	1.06 (1.04–1.08)	<0.001
180-day mortality						
ePVS	1.14 (1.12–1.16)	<0.001	1.08 (1.07–1.10)	<0.001	1.07 (1.05–1.09)	<0.001
Renal function recovery rate						
ePVS	0.91 (0.89–0.93)	<0.001	0.95 (0.93–0.97)	<0.001	0.96 (0.94–0.99)	0.002

Crudel model: Unadjusted.

Model 1: Adjust for Age, Race, Sex, Weight, Congestive heart failure, COPD, Cancer, Diabetes, RRT, Vasoactive Drug Support, renal failure, AKI stage.

Model 2: Adjust for ePVS, Age, Race, Sex, Weight, Congestive heart failure, COPD, Cancer, Diabetes, RRT, Vasoactive Drug Support, renal failure, AKI stage, Glucose, INR, PT, APTT, WBC, Platelets, Na, K, Ca, Cl, Creatinine, BUN, Heart rate, SBP, DBP, Respiratory rate, Temperature, SpO2, SOFA, SAPSII, GCS.

Logistic regression analysis further revealed that ePVS was negatively correlated with the probability of renal function recovery. In the unadjusted model, the odds ratio (OR) was 0.91 (95% CI 0.89–0.93, *p* < 0.001). In Model 2, the OR was 0.95 (95% CI 0.93–0.97, *p* < 0.001), and in Model 3, the OR was 0.96 (95% CI 0.94–0.99, *p* = 0.002).

### RCS analysis

**Figure 2.**
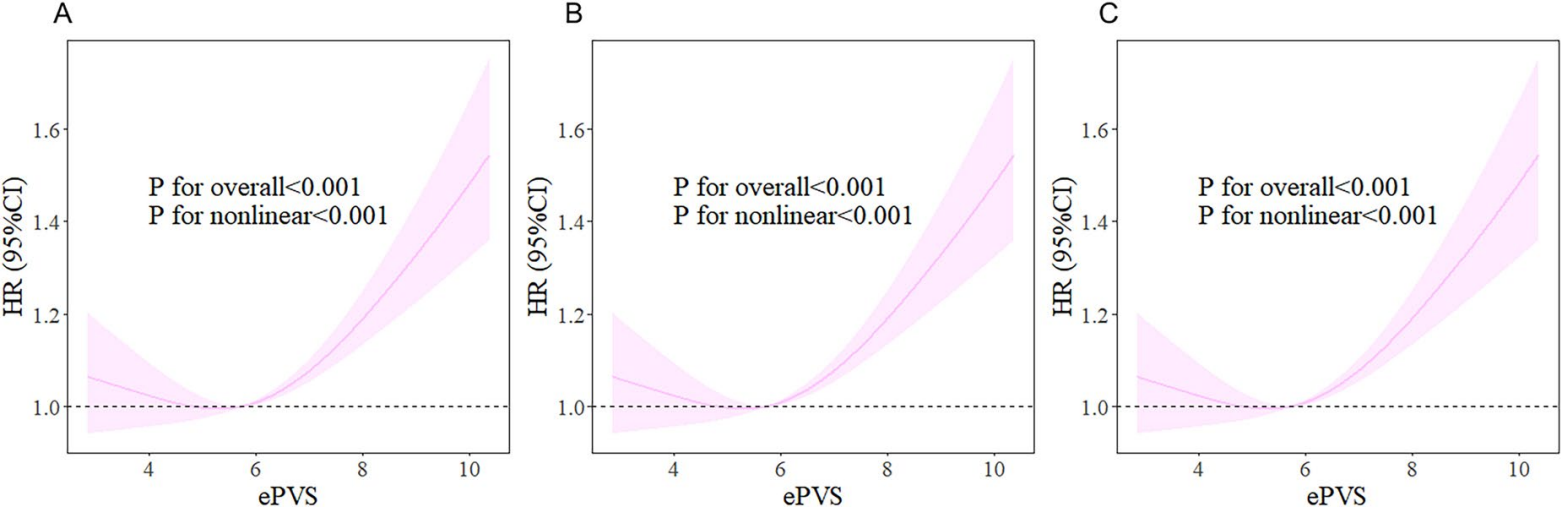
Restricted cubic spline curve depicting the association between ePVS and 28-Day (A), 60-Day (B), and 180-Day (C) Mortality in SA-AKI patients.

We employed the RCS model to investigate the potential nonlinear relationship between ePVS and 28-day mortality. The optimal configuration for the restricted cubic spline was identified as three knots. The detailed selection process is summarized in Table S2. As shown in Figure 2, the analysis revealed a significant U-shaped nonlinear relationship between ePVS and 28-day mortality in the fully adjusted model (nonlinear *p* < 0.001), which was also evident for 60-day and 180-day mortality. Further threshold effect analysis, detailed in Table 3, identified an ePVS threshold of 5.47. Above this threshold, the risk of mortality significantly increased with higher ePVS levels (HR 1.10, 95% CI 1.07–1.13, *p* < 0.001). Conversely, below this threshold, the risk of death decreased significantly as ePVS levels rose (HR 0.92, 95% CI 0.87–0.97, *p* = 0.003).

**Table 3.** Threshold effect analysis of ePVS on 28-day mortality in SA-AKI patients.

28-day mortality	The effect size, HR (95%CI)	*P* value
Fitting model by standard linear regression	1.05 (1.03–1.07)	<0.001
Fitting model by two-piecewise linear regression		
Inflection point	5.47	
ePVS < 5.47	0.92 (0.87–0.97)	0.003
ePVS > 5.47	1.10 (1.07–1.13)	<0.001
*P* for likelihood ratio test		<0.001

### Correlation between ePVS and prognosis in different groups

Based on the ePVS threshold, we classified patients into two groups: a high ePVS group (ePVS ≥ 5.47) and a low ePVS group (ePVS < 5.47). As summarized in Table 4, the high ePVS group exhibited significantly higher mortality at 28, 60, and 180 days, along with a lower proportion of renal function recovery compared to the low ePVS group. Further analysis in the fully adjusted Model 3 (Table 5) showed that the high ePVS group had a significantly increased risk of 28-day mortality (HR 1.09, 95% CI 1.02–1.17, *p* < 0.001), 60-day mortality (HR 1.12, 95% CI 1.05–1.20, *p* < 0.001), and 180-day mortality (HR 1.15, 95% CI 1.09–1.22, *p* < 0.001). Additionally, the high ePVS group had a significantly lower rate of renal function recovery (OR 0.87, 95% CI 0.81–0.94, *p* < 0.001).

**Table 4.** Differences in prognostic outcomes between high and low ePVS groups.

	ePVS < 5.47	ePVS ≤ 5.47	P-value
28-day mortality	20%	25%	<0.001
60-day mortality	24%	31%	<0.001
180-day mortality	29%	38%	<0.001
Renal function recovery rate	73%	67%	<0.001

**Table 5.** Association between different levels of ePVS and prognosis in SA-AKI patients.

	Crude model		Model 1		Model 2	
	95%CI	P	95%CI	P	95%CI	P
28-day mortality						
ePVS <5.47	ref		ref		ref	
ePVS ≥ 5.47	1.27 (1.19–1.36)	<0.001	1.1 (1.03–1.18)	0.01	1.09 (1.02–1.17)	0.01
60-day mortality						
ePVS <5.47	ref		ref		ref	
ePVS ≥ 5.47	1.31 (1.24–1.40)	<0.001	1.13 (1.06–1.20)	<0.001	1.12 (1.05–1.20)	<0.001
180-day mortality						
ePVS <5.47	ref		ref		ref	
ePVS ≥ 5.47	1.37 (1.29–1.44)	<0.001	1.15 (1.08–1.22)	<0.001	1.15 (1.09–1.22)	<0.001
Renal function recovery rate						
ePVS <5.47	ref		ref		ref	
ePVS ≥ 5.47	0.75 (0.70–0.80)	<0.001	0.85 (0.79–0.92)	<0.001	0.87 (0.81–0.94)	<0.01

Crudel model: Unadjusted.

Model 1: Adjust for Age, Race, Sex, Weight, Congestive heart failure, COPD, Cancer, Diabetes, RRT, Vasoactive Drug Support, Renal failure, AKI stage.

Model 2: Adjust for ePVS, Age, Race, Sex, Weight, Congestive heart failure, COPD, Cancer, Diabetes, RRT, Vasoactive Drug Support, Renal failure, AKI stage, Glucose, INR, PT, APTT, WBC, Platelets, Na, K, Ca, Cl, Creatinine, BUN, Heart rate, SBP, DBP, Respiratory rate, Temperature, SpO2, SOFA, SAPSII, GCS.

**Figure 3.**
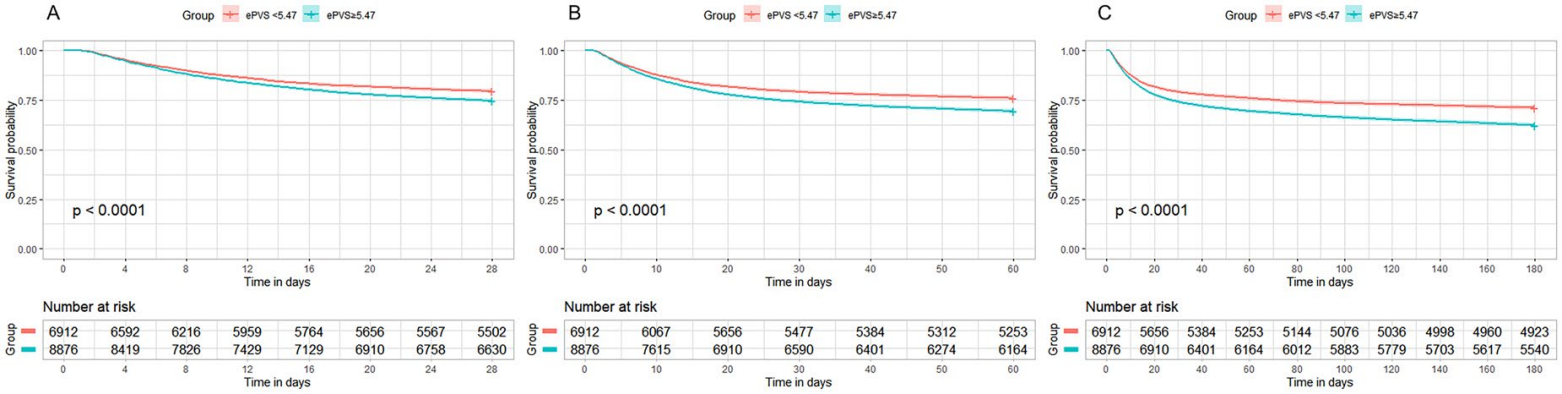
Kaplan-Meier survival curves for SA-AKI patients with high and low ePVS levels at 28 Days (A), 60 Days (B), and 180 Days (C).

As depicted in Figure 3, the Kaplan-Meier curves demonstrated that the high ePVS group had significantly lower cumulative survival rates at 28, 60, and 180 days compared to the low ePVS group (*p* < 0.001). These findings suggest that elevated ePVS levels are closely associated with poor prognosis in patients with SA-AKI.

### Subgroup analysis

**Figure 4.**
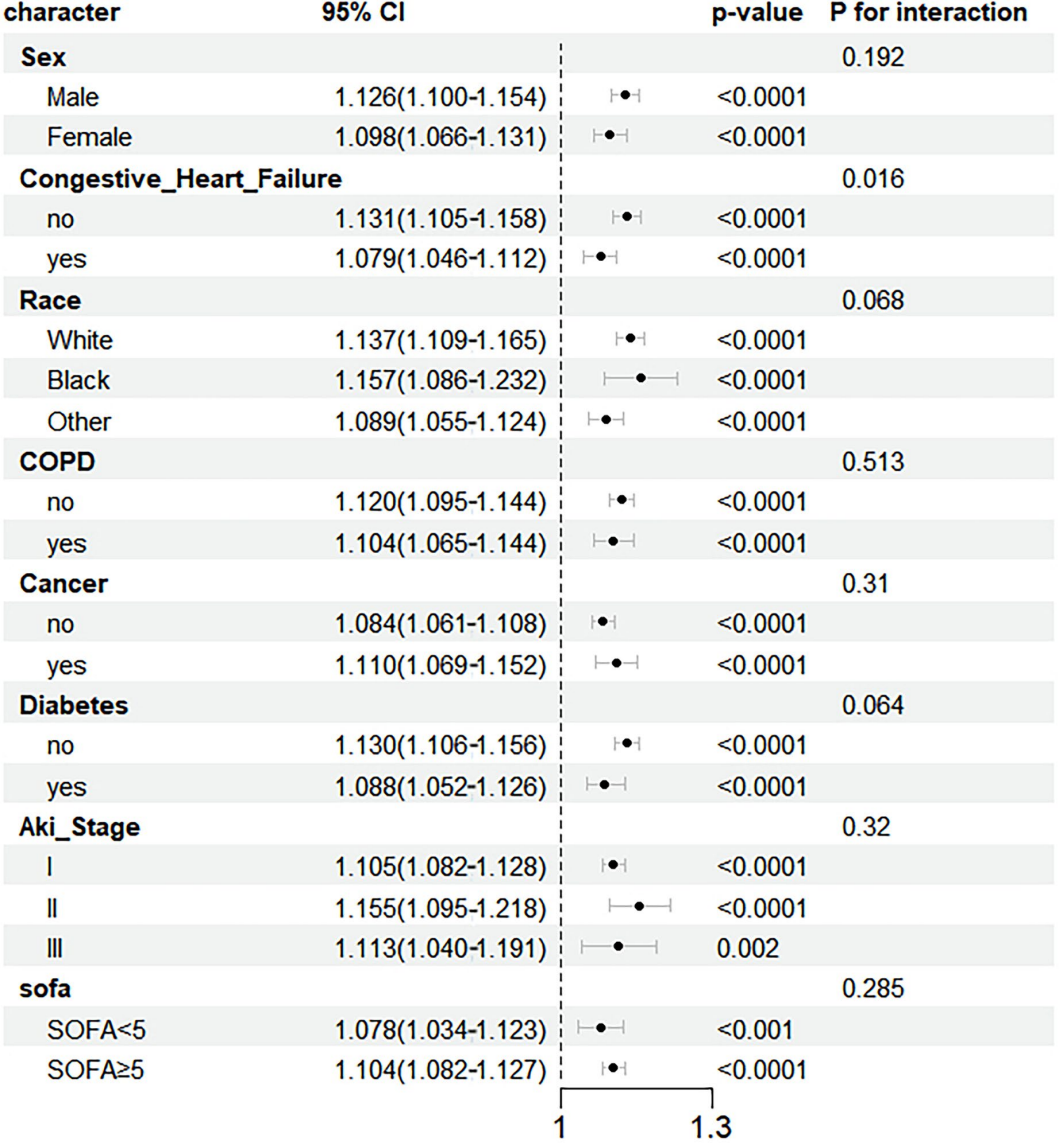
Subgroup analysis of the association between ePVS and 28-day mortality in SA-AKI patients. Forest Plot of subgroup analysis. Note: Cox regression analysis was performed to evaluate the association between ePVS and survival outcomes within each subgroup. Results are reported as HR with 95% CI. Interaction p-values were calculated using Cox regression to determine whether interactions existed between subgroups and survival outcome.

Subgroup analysis was conducted to assess the stability of the relationship between ePVS and 28-day mortality in SAAKI patients (Figure 4). No significant interaction was observed between ePVS and 28-day mortality in the subgroups of sex, race, COPD, AKI staging, diabetes, and SOFA scores (P interaction > 0.05). Although interaction effects were observed in the heart failure and kidney disease subgroups, the direction of the effects remained consistent across all subgroups. In the heart failure subgroup, the HR was 1.131 (95% CI 1.105–1.58), compared to 1.079 (95% CI 1.046–1.112) in the non-heart failure subgroup. In the kidney disease subgroup, the HR was 1.132 (95% CI 1.107–1.157), whereas in the non-kidney disease subgroup, it was 1.041 (95% CI 1.004–1.079).

## Discussion

This study is the first to investigate the relationship between ePVS and prognosis in ICU patients with SA-AKI. Our findings reveal a significant U-shaped nonlinear relationship between ePVS and 28-day mortality. Further threshold effect analysis indicated that ePVS levels above 5.47 dL/g were positively associated with an increased risk of death, whereas ePVS levels below 5.47 dL/g showed an inverse relationship with mortality risk. These associations remained robust even after adjusting for multiple potential confounders, suggesting that ePVS is an independent predictor of mortality and renal function recovery and providing valuable insights for risk assessment in SA-AKI patients.

PV plays a crucial role in regulating the balance between interstitial and intravascular fluids [27]. Estimating plasma volume through a simple formula based on hemoglobin and hematocrit concentrations provides an effective method for monitoring fluid status [28]. Previous studies have shown that ePVS has demonstrated strong prognostic value in various cardiovascular conditions. Nogi et al. [20] investigated the relationship between ePVS and long-term outcomes in acute myocardial infarction patients. Those with high ePVS had significantly higher rates of all-cause mortality, cardiovascular mortality, and heart failure hospitalizations. Even after adjusting for confounders, high ePVS was associated with a 1.9-fold increase in all-cause mortality. Additionally, in a post-hoc analysis of the EPHESUS study [18], researchers found that the predictive value of ePVS for early cardiovascular events surpassed that of conventional clinical assessments. The kidneys, being particularly sensitive to changes in blood volume, are notably affected by fluid overload. Several studies have examined the relationship between ePVS and the risk of AKI. Yang et al. [29] assessed the correlation between ePVS and AKI risk in patients undergoing coronary revascularization and found that higher ePVS was associated with a greater likelihood of AKI (OR = 1.06, 95% CI: 1.02–1.10). Similarly, Isha et al. [30] calculated plasma volume using ePVS and PVS formulas in patients with COVID-19-related acute respiratory distress syndrome and found that both ePVS (OR = 1.162, 95% CI: 1.048–1.288, *p* = 0.004) and PVS (OR = 1.032, 95% CI: 1.012–1.050, *p* = 0.001) were independent predictors of new-onset AKI.

In patients with SA-AKI, the immediate administration of intravenous fluids is critical for restoring intravascular volume and maintaining adequate tissue perfusion and oxygen delivery [4,31]. Therefore, volume assessment plays a vital role in fluid management for these patients. Current sepsis management guidelines recommend resuscitation with a rapid fluid bolus (at least 30 mL/kg of body weight in the first 3 h) [32]. However, both excessive and inadequate resuscitation are associated with poor outcomes [32]. Several studies have shown that a positive fluid balance after AKI is a key factor contributing to the increased mortality in SA-AKI patients [6,7]. Factors such as endothelial dysfunction, impaired cardiac output, increased cardiac preload, and intra-abdominal pressure due to tissue edema can worsen the condition of SA-AKI patients by impairing renal venous return, leading to fluid overload [33,34]. In our study, we identified a significant U-shaped nonlinear relationship between ePVS and mortality. ePVS was negatively associated with SA-AKI outcomes when below the threshold of 5.47 dL/g, but positively associated with mortality risk when above this threshold. Consistent with the mortality findings, the relationship between ePVS and renal recovery followed the same pattern, with ePVS below 5.47 dL/g associated with a higher likelihood of renal function recovery. Our finding of a U-shaped relationship between ePVS and mortality is notably consistent with recent literature on other severe infections. For instance, a study of patients with COVID-19 similarly reported that both low and high ePVS levels were associated with increased mortality risk [35]. This parallel observation across different disease entities suggests that the U-shaped relationship may be a fundamental characteristic of dysregulated volume status in critical illness, rather than a phenomenon unique to SA-AKI. It underscores the universal clinical challenge of achieving optimal volume homeostasis. This relationship likely reflects the dual impact of over-resuscitation and under-resuscitation on patient outcomes. The ePVS threshold of 5.47 dL/g may serve as a critical point for clinicians when performing fluid resuscitation in patients with SA-AKI. Exceeding this threshold warrants particular attention to venous return function during fluid therapy and vigilance for potential fluid overload. In subgroup analyses, significant interactions were observed between ePVS and both HF and CKD. The association of ePVS with 28-day mortality was more pronounced in patients without HF or CKD, likely reflecting differences in underlying volume status and compensatory reserve. In patients with HF or CKD, chronic volume overload and diminished regulatory capacity result in a higher baseline ePVS, thereby diluting the prognostic impact of any further acute increase which becomes masked by the underlying high-risk state. In contrast, among those without HF or CKD, an rise in ePVS may more accurately signal acute volume decompensation, thereby strengthening its effect on mortality.

Our study also has some limitations. First, as a retrospective cohort study, the influence of unmeasured confounders could not be entirely excluded, despite adjusting for potential confounders in our models. Second, the data were obtained from a single-center cohort, which may limit the generalizability of our findings. Future prospective, multicenter, high-quality studies are needed to further validate our results. Third, this study used the MIMIC-IV database, which lacked detailed echocardiographic and hemodynamic data, limiting our ability to conduct a more in-depth analysis of the relationship between volume metrics and ePVS. Fourth, it should be noted that the ePVS, derived from hemoglobin and hematocrit, may have reduced accuracy in patients with extreme anemia. Therefore, the interpretation of ePVS values in such populations should be made with caution. Furthermore, the Strauss-Duarte formula may systematically underestimate true plasma volume [36], a bias that should be considered when interpreting absolute ePVS values. Finally, the findings from our subgroup analyses, particularly the interaction effects, should be interpreted with caution. These results are exploratory and require further validation in independent prospective cohorts to confirm their robustness.

### Conclusion

This study demonstrates that ePVS is strongly associated with poor prognosis in ICU patients with SA-AKI. It serves as an independent risk factor for 28-day mortality and exhibits a U-shaped nonlinear relationship with death risk. Larger prospective cohort studies or randomized controlled trials are needed to further validate the prognostic value of ePVS in SA-AKI. Additionally, future research should explore whether adjusting fluid management strategies based on ePVS levels can improve patient outcomes.

## Supplementary Material

Supplementary Table.docx

## Data Availability

Our data was obtained from MIMIC-IV3.1. This data can be found here: MIMIC-IV v3.1 (physionet.org), thus no more permission was required.
