# Supplementary material for: Association between estimated plasma volume status and risk of sepsis-associated acute kidney Injury: a retrospective cohort study using the MIMIC-IV database
Source: Ren Fail. 2025 Nov 14;47(1):2586382. doi: 10.1080/0886022X.2025.2586382 (PMC12621331; doi:10.1080/0886022X.2025.2586382)
Supplement: Supplementary Table.docx [file IRNF_A_2586382_SM4636.docx]

**TableS1 Variance inflation factor (VIF) analysis of Cox regression model variables for 28-day mortality in ePVS and SAAKI patients**

| **Variable** | **VIF** |
| --- | --- |
| Age | 1.81933 |
| RaceOther | 3.259287 |
| RaceWhite | 3.311932 |
| GenderM | 1.117179 |
| Weight | 1.209486 |
| Congestive.heart.failureyes | 1.250939 |
| COPD yes | 1.060889 |
| Cancer yes | 1.147444 |
| Diabetes yes | 1.238934 |
| RRT yes | 1.583995 |
| Vasoactive. Drug yes | 1.525079 |
| Renal.failure yes | 1.412749 |
| aki_stage_prime2 | 1.035574 |
| aki_stage_prime3 | 1.118041 |
| Glu | 1.210026 |
| INR | 6.809242 |
| PT | 6.891549 |
| APTT | 1.122473 |
| WBC | 1.095953 |
| Platelets | 1.212134 |
| Na | 2.408198 |
| K | 1.244634 |
| Ca | 1.133384 |
| Cl | 2.576117 |
| BUN | 2.293621 |
| Creatinine | 2.739195 |
| Heart.rate | 1.298908 |
| SBP | 1.460836 |
| DBP | 1.479074 |
| Respiratory.rate | 1.160866 |
| Temperature | 1.134282 |
| SpO2 | 1.079328 |
| SOFA | 2.914122 |
| SAPSII | 3.493386 |
| GCS | 1.624745 |

**TableS2 Comparison of Model Fit Indices for Restricted Cubic Spline Models with varying Knot Numbers for ePVS**

| **Number of Knot** | **AIC** | **BIC** | **Likelihood Ratio Chi-Square** | **R2** | **Dxy** |
| --- | --- | --- | --- | --- | --- |
| **3** | **67598.23** | **67858.91** | **2253.74** | **0.135** | **0.437** |
| **4** | **67599.6** | **67867.95** | **2254.36** | **0.135** | **0.437** |
| **5** | **67601.35** | **67877.36** | **2254.62** | **0.135** | **0.437** |
| **6** | **67602.04** | **67885.72** | **2255.93** | **0.135** | **0.437** |
